# An InSAR Interferogram Filtering Method Based on Multi-Level Feature Fusion CNN

**DOI:** 10.3390/s22165956

**Published:** 2022-08-09

**Authors:** Wang Yang, Yi He, Sheng Yao, Lifeng Zhang, Shengpeng Cao, Zhiqing Wen

**Affiliations:** 1 Faculty of Geomatics, Lanzhou Jiaotong University, Lanzhou 730070, China; 2 National-Local Joint Engineering Research Center of Technologies and Applications for National Geographic State Monitoring, Lanzhou 730070, China; 3 Gansu Provincial Engineering Laboratory for National Geographic State Monitoring, Lanzhou 730070, China

**Keywords:** interferometric synthetic radar (InSAR), interferogram filtering, convolutional neural network (CNN), feature learning

## Abstract

Interferogram filtering is an essential step in processing data from interferometric synthetic aperture radar (InSAR), which greatly improves the accuracy of terrain reconstruction and deformation monitoring. Most traditional interferogram filtering methods achieve noise suppression and detail preservation through morphological estimation based on the statistical properties of the interferogram in the spatial or frequency domain. However, as the interferogram’s spatial distribution is diverse and complex, traditional filtering methods struggle to adapt to different distribution and noise conditions and cannot handle detail preservation and noise suppression simultaneously. The study proposes a convolutional neural network (CNN)-based multi-level feature fusion model for interferogram filtering that differs from the traditional feedforward neural network (FNN). Adopting a multi-depth multi-path convolution strategy, the method preserves phase details and suppresses noise during interferogram filtering. In filtering experiments based on simulated data, qualitative and quantitative evaluations were used to validate the performance and generalization capabilities of the proposed method. The method’s applicability was evaluated by visual observation during filtering and unwrapping experiments on real data, and the time-series deformation acquired by time series (TS)-InSAR technique is used to evaluate the effect of interferogram filters on deformation monitoring accuracy. Compared to commonly used interferogram filtering methods, the proposed method has significant advantages in terms of performance and efficiency. The study findings suggest new directions for research on high-precision InSAR data processing and provide technical support for practical applications of InSAR.

## 1. Introduction

Interferometric synthetic aperture radar (InSAR) is a high-precision microwave interferometry technology for three-dimensional wide-area terrain reconstruction and deformation monitoring [1,2,3,4] based on conjugate multiplication of two complex SAR images. However, the inherent characteristics of the SAR imaging system mean that InSAR interferograms are seriously compromised by spatiotemporal decoherence, atmospheric delay, system thermal noise, and other factors that undermine the accuracy of terrain or deformation inversion results [5,6]. Interferogram filtering to suppress noise and preserve detailed information improves the accuracy of terrain reconstruction for three-dimensional and deformation monitoring and is critical for InSAR inversion accuracy.

The traditional interferogram filtering methods can be divided into spatial domain filtering and frequency domain filtering [7]. In spatial domain filtering, defined (linear or nonlinear) operations, such as the Pivoting median filter [8], Lee filter [9], NL-InSAR [10], and InSAR-BM3D are performed on the neighborhood pixels of the phase images to complete image smoothing and denoising [11]. The Pivoting median filter uses sliding convolution templates for interferogram denoising in complex domains. The Pivoting median filter performs well in regions with small phase gradients and high coherence, but this algorithm inevitably suffers from reduced spatial resolution and inadequate denoising and detail preservation in low-coherence regions [12]. The Lee filter is a directional window-based method and calculates the local noise variance in the selected directional window to perform adaptive filtering. The Lee filter overcomes the shortcomings of regular rectangular windows. However, in the low-coherence regions, the direction of the filter window is often difficult to judge accurately, and the fringe structure may be damaged after filtering. The size of the Lee filter’s window is fixed, and the direction is finite, which means the filter cannot capture fringe direction using a predefined window in regions where fringe direction changes rapidly [13]. The NL-InSAR filter is based on maximum likelihood estimation, this processing strategy can capture global distribution characteristics and has better adaptive capability. However, the NL-InSAR filter leads to a noticeable increase in computational effort and a significant decrease in efficiency [14]. In subsequent research, the BM3D algorithm was applied to InSAR interferogram filtering. InSAR-BM3D offers the performance advantages of non-local filtering and better resolution in wavelet domain filtering. However, the inefficiency of InSAR-BM3D and the marginal improvement of detail preservation in low-coherence regions make it difficult to use in large-scale or complex terrain [15]. As for frequency domain filtering, the most classical algorithm is the Goldstein filter [16,17], it transform the phase into the frequency domain by Fourier transform and performs thresholding or weighting according to the frequency distribution of the noise and phase information to achieve noise suppression and detail preservation [18,19,20]. The Goldstein filter is the first frequency domain method to be applied to interferograms. In essence, this method weights the two-dimensional spectrum of the interferogram by thresholding and shrinking its magnitude, but the defects of its filtering parameters limit the algorithm’s performance. First, its filtering parameters can only be taken from 0 to 1, which is underperforming for low-coherence regions. Second, as parameter values are preset and remain fixed while interferogram noise is spatially variable, high-coherence regions will be over-filtered, and low-coherence regions will be under-filtered [16]. 

In recent years, the InSAR interferogram filtering technology has entered the era of deep learning. Using the original image as input, a convolutional neural network (CNN) extracts a range of abstract features from that image by means of a simple non-linear model, enabling fast and accurate image reconstruction. Among the existing CNN-based filtering models, interferogram denoising convolutional neural network (IPDnCNN) and Φ-Net have a significant improvement in performance [21,22,23], and to a certain extent, they solve the problems faced by the above traditional methods. IPDnCNN adopts the traditional feedforward sequential convolutional neural network and removes the noise from the interferogram to obtain the filtered phase through residual learning. This method can reduce the phase noise while protecting fringe edges and avoiding the use of filter windows, and far outperforms traditional filtering methods in performance. However, in the feature extraction process, this feed-forward sequence structure tends to make the model lose low-level features. The lost features contain a large amount of detailed information, such as structure and location. Therefore, preserving the low-level features and performing feature decoding is necessary to improve the detail preservation capability of the interferogram filtering networks. Φ-Net replaces the single U-Net layers with residual blocks (RBs), the cascade of encoder and decoder stages used in combination with skip connections and residual shortcuts enables an effective representation of the interferometric signal and the superimposed noise. The Φ-Net framework is a multi-scale feature fusion network structure, which takes into account the ability of U-Net to restore information at different scales and the generalization ability of the model applied to the real data. However, the feature fusion strategy of Φ-Net method is the same-depth merging of encoder and decoder features, and this hopping connection tends to make the network suffer from the semantic gap during feature extraction [24].

According to the above, the problems currently faced by existing interferogram filtering methods can be roughly summarized as follows: (1) Noise suppression is insufficient and detail preservation is limited, especially in areas with low-coherence and dense fringes; (2) Model generalization ability is poor, and the filtering performance depends strongly on the noise level; (3) The huge number of parameters of previous deep learning methods makes the model very slow in fitting, increasing the time and hardware cost of model training.

To solve the above problems, we proposed a CNN-based method of interferogram filtering based on feature extraction and image reconstruction under supervised conditions. Firstly, in the network design, the multi-scale feature fusion strategy in Φ-Net was adapted and the hopping connection structure was modified into a progressive connection structure, which has the advantages of making the model more comprehensive in taking different scales into account, reducing the information loss in the convolution process, and enhancing the noise suppression and detail retention of the model in interferogram filtering. Secondly, to improve the structural accuracy and numerical accuracy of the output image, the loss function of the joint structural similarity (SSIM) and mean absolute error (MAE) was adopted [25,26], the SSIM was responsible for controlling the structural accuracy of the image and MAE was responsible for controlling the numerical accuracy of the image. Combined with the loss function described above, the network used direct output with the activation function of “Tanh” rather than residual learning, which improves the generalization ability and self-adaptability of the model. In addition, the convolution layer was used before the fusion of the feature maps at each scale, which makes the model more lightweight, compared to Φ-Net, the number of parameters is reduced significantly (from roughly 10 million to 2 million) with similar feature map size, which makes the model easier to fit and shortens the training time.

The objectives of the study were as follows. (1) To build a multi-level feature fusion network by integrating convolutional layers of different depths in the channel dimension to form multi-level features. The multi-level feature map was then used for feature decoding and image reconstruction. (2) To evaluate the applicability and performance of the proposed method in simulated and real data-based experiments, and to compare it with the pivoting median filter, Lee filter, Goldstein filter, NL-InSAR, InSAR-BM3D and Φ-Net.

## 2. Methods

### 2.1. Proposed Model Network Architecture

**Figure 1:**
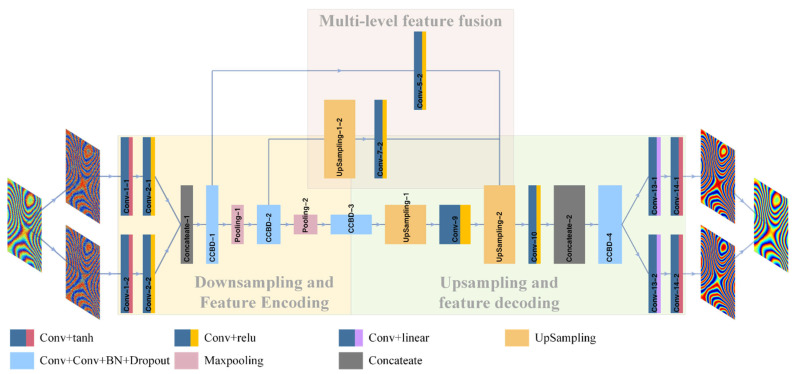
Proposed network model architecture.

For the purposes of this study, we developed an end-to-end CNN model for InSAR interferogram filtering. The network structure (Figure 1) was based on a multi-level feature fusion strategy involving three main processes: (1) downsampling and feature encoding; (2) upsampling and multi-level feature fusion; and (3) image reconstruction and generation. 

#### 2.1.1. Downsampling and Feature Encoding

In downsampling and feature encoding for interferogram feature extraction, 2D convolution was first used to extract features from the real and imaginary parts of the interferogram. The features were then integrated by the concatenate layer. Finally, the features were encoded by progressive downsampling, using 2D convolution, maximum pooling, batch normalization (BN), and dropout layers [27,28]. For this process, all convolution kernels were sized 3 × 3. To ensure that the size of the output feature map was the same as the current one, the edges of the feature map were designated “same”. As the size of the feature map decreased, the output channel of the convolution layer increased exponentially from a feature map size of 32 to 128 and 256. In the pooling process, the window under the maximum-size feature map was 2 × 2, with a step size of 2. The feature map filling method was “valid” for all pooling layers to ensure that pixel values were not repeatedly computed while all pixels were traversed, and feature map width and height were reduced to the desired size.

When training a deep network, fitting slows down and requires a slower learning rate because internal covariate shift (ICS) [29] reduces the model’s generalization capability and increases time consumption. As BN can resolve this problem, we used BN layers after two convolutions in CCBD-i {i = 1, 2, 3, 4} (two convolutions, one batch normalization, and one dropout) to accelerate network training and to improve the model’s generalization ability. Based on the existing literature and extensive experimental evidence, adding the dropout layer after the BN layer prevented model overfitting by discarding neurons with a probability of 0.25.

#### 2.1.2. Upsampling and Multi-Level Feature Fusion

The upsampling and multi-level feature fusion process utilized multi-layer and multi-path upsampling and convolution to generate feature maps at different scales; using the concatenate layer, these were then stitched together in the channel dimension. Path 1 started from CCBD-1 and used one convolution (Conv-5-2) to generate the first level of the feature map, which did not change in size for this path. Path 2 started at CCBD-2 and underwent upsampling (UpSampling-1-2) and convolution (Conv-7-2) to generate the second level of the feature map, which increased in size from 128 × 128 to 256 × 256. Path 3 started at CCBD-3 and went through UpSampling-1 and Conv-9. After securing the three feature map levels, concatenate-2 was used to stitch the different levels together in the channel dimension to produce a fusion feature that captured both deep and shallow features.

In this process, the sampling factor for the three upsamples was 2 for both rows and columns, and the interpolation method was “nearest”. To reduce the hard-edge problem caused by the nearest neighbor algorithm in the upsampling layer, the kernel size of the convolutional layer connected after each upsampling layer was the same as the upsampling factor (2 × 2).

#### 2.1.3. Image Reconstruction and Generation

The purpose of the image reconstruction and generation process is to split the features and restore the real and imaginary images, gradually restoring the details of the image. In CCBD-4, the convolution kernel size was 3 × 3; after the double-output split, the two convolution kernels of 2 × 2 and 1 × 1, respectively, gradually reduced the feature dimension and recovered details of the image’s real and imaginary parts.

### 2.2. Model Training

All of the experiments in this study were performed on a computer with an Intel Core i7-10700F CPU and an NVIDIA GeForce GTX 1080ti GPU. After many experiments, and taking account of model calculation efficiency, result accuracy, and hardware conditions, the final training parameters set the epoch to 128 and the batch size to 12. Adaptive motion estimation (Adam) was used as the optimizer [30], and the initial learning rate was set to 10^−4^. Because the feature map stored temporarily during calculation was large, a half-precision (16-bit) floating-point format model was used for training. In the gradient descent algorithm, the index for evaluating pixel value difference or similarity of image structure could not preserve the details of the interferogram while simultaneously maintaining resolution. We used the custom loss function L(Θ), which is a weighted combination of MAE SSIM based on the following formula:

(1) $$L(\Theta )=W_{MAE}\times MAE+W_{SSIM}\times SSIM$$

where Θ refers to trainable parameters of the proposed model**;** $W_{MAE}$ and $W_{SSIM}$ are the weights of MAE and SSIM. In the present experiment, $W_{MAE}=0.5$, $\mathrm{and}\;W_{SSIM}=0.5.$

MAE is calculated as

(2) $$MAE=\frac{1}{n}\sum_{i=1}^{n}\left|{\hat{\varphi }}_{i}-\varphi_{i}\right|$$

where *n* is the number of samples, and ${\hat{\varphi }}_{i}$ and $\varphi_{i}$ are the filtered and clean images, respectively.

SSIM is calculated as

(3) $$SSIM(\varphi ,\hat{\varphi })=\frac{\left(2\mu_{\varphi_{i}}\mu_{{\hat{\varphi }}_{i}}+{\left(k_{1}L\right)}^{2})(2\sigma_{\varphi_{i}{\hat{\varphi }}_{i}}+{\left(k_{2}L\right)}^{2}\right)}{\left(\mu_{\varphi_{i}}^{2}+\mu_{{\hat{\varphi }}_{i}}^{2}+{\left(k_{1}L\right)}^{2})(\sigma_{\varphi_{i}}^{2}+\sigma_{{\hat{\varphi }}_{i}}^{2}+{\left(k_{2}L\right)}^{2}\right)}$$

where $\mu_{\varphi }$ and $\mu_{\hat{\varphi }}$ are the mean of the clean image and the filtered image, respectively, and $\sigma_{\varphi }$ and $\sigma_{\hat{\varphi }}$ are their standard deviations; $\sigma_{\varphi \hat{\varphi }}$ is the covariance of the clean and the filtered image phase matrix; L is the dynamic range of the phase images; $k_{1}$ and $k_{2}$ are constants (by default, $k_{1}=0.01$ and $k_{2}=0.03$; their function is to prevent an unstable result caused by an unduly small denominator).

## 3. Results

To evaluate the performance of the proposed method, we performed a series of experiments using simulated and real InSAR interferograms, using the pivoting median, Lee, Goldstein, NL-InSAR, and InSAR-BM3D filters for comparison purposes. The main method of evaluation was qualitative, based on visual observation; for supplementary quantitative evaluation and validation, we employed RMSE and SSIM.

### 3.1. Experimental Data Set

A large number of interferogram pairs (noisy and noise-free) were needed for model training and testing. However, as the interferogram is difficult to construct from SAR images [31], we simulated noisy and noise-free interferograms that were similar to the real interferogram in terms of distribution. These simulated cases included two sources: simulated terrain and digital elevation models (DEM). Simulated terrain topography is smoother and more stable, and these interferograms provide generalized scenarios for model training [32]. DEM is closer to real topography, and these interferograms provided specialized scenarios [33]. In total, 5000 interferogram pairs were generated, with a dataset ratio of 1:1 (simulated terrain to DEM). For the purposes of the study, the designated ratio of training sets to testing sets was 4:1.

### 3.2. Experiment Using Simulated Data

**Figure 2:**
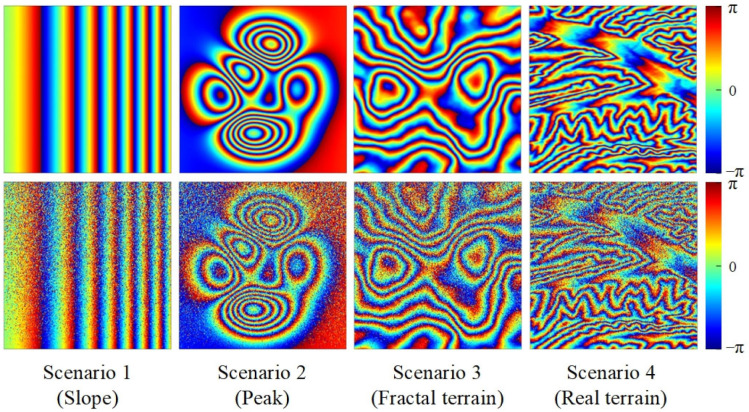
Four simulated interferograms with different distribution characteristics ranging from no noise (top row) to fading noise (bottom row).

Four distinct types of simulated interferogram (Figure 2) were selected for the simulated data experiments.

- Scenario 1 (Slope): the interferogram was generated from simulated terrain of varying gradient, with fringes spacing from 50 to 24 pixels.
- Scenario 2 (Peak): a mountain-like interferogram was generated by summing Gaussian surfaces with different means and variances.
- Scenario 3 (Fractal terrain): the interferogram was generated from random topography using fractal mathematics.
- Scenario 4 (Real terrain): the interferogram was synthesized using a real DEM, which was randomly cropped from a 30 resolution SRTM DEM of China.

In these scenarios, the coherence of the four simulated interferograms varied gradually from 0.2 (top of images) to 0.7 (bottom of images), the change in coherence can be used to evaluate the dependence of the filtering performance on the noise intensity.

**Figure 3:**
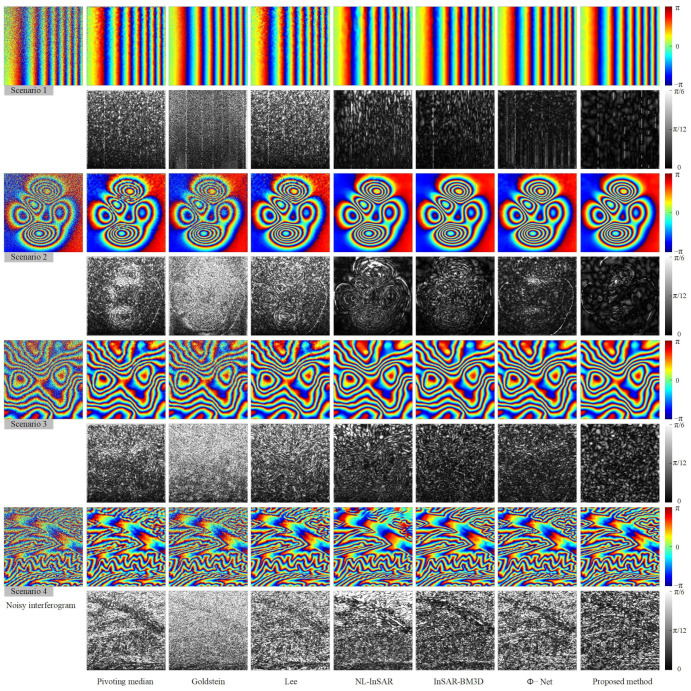
Noisy interferograms and filtered interferograms for the four simulation scenarios. The false color images are the noisy or filtered interferograms, and the black and white images are the phase error images corresponding to the false color image above. The phase error image is the difference image between the filtered and the noiseless interferogram, the white textures in the phase error images represent the error in the phase filtering.

**Figure 4:**
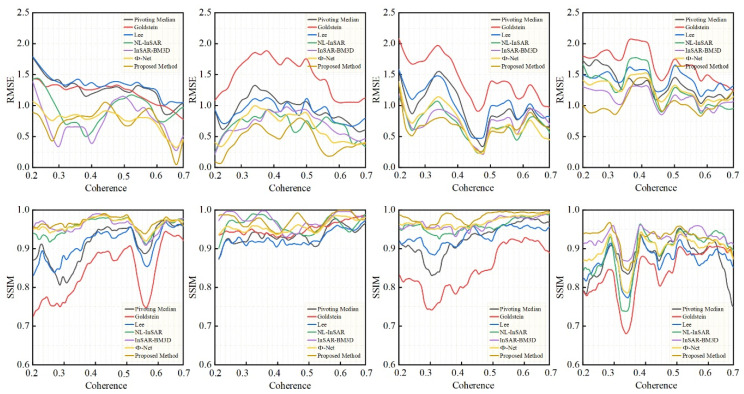
RMSE and SSIM variation curves for the four simulated interferograms (left to right): scenarios 1–4.

We conduct qualitative and quantitative evaluation of the performance of filtering methods from the four scenarios set. Figure 3 shows the qualitative evaluation results of different filtering methods in different scenarios, and Figure 4 shows the evaluation results of quantitative indicators.

Qualitative evaluation analysis was as follows:

In Scenario 1 (the first and second lines in Figure 3), the variation of fringe density, coherence has different degrees of influence on the performance of different filtering methods. As can be seen from Figure 3, the filtered interferograms of the Pivoting median filter and Lee filter show a significant resolution loss. The Goldstein filter do not destroy the fringe structure, but the denoising ability is limited, and the fringe edges are very blurred after filtering, fewer dark black areas in the phase error image, indicating that the adaptive ability of the Goldstein filter is insufficient. NL-InSAR and InSAR-BM3D exhibit good noise suppression, but after NL-InSAR and InSAR-BM3D filtering, the low coherence region recovers curved fringe edges, which is obviously incorrect, and there is also a large amount of white phase error in the phase error images. Both deep learning methods (Φ-Net and proposed methods) perform very well in Scenario 1, with the phase error images appearing essentially uniformly black, in contrast, the proposed method has flatter, cleaner fringes and provide a phase error image that contains less error. As the fringe density and coherence change, the proposed methods are able to effectively cope.

In Scenario 2 (the third and fourth lines in Figure 3), annular fringes with different densities could be used to effectively evaluate the fringe structure and phase edge preservation ability of different filtering methods. In the filtered image, the Pivoting median filter, Lee filter and Goldstein filter methods show severe fringe structure damage in the regions with dense fringes, and in the phase error image. In contrast, the proposed method has the least significant annular error. Small areas of structure damage also appear in the locations with very dense fringes, but this error can be accepted considering the superior filtering performance in other regions.

In Scenario 3 (the fifth and sixth lines in Figure 3), there are both circular fringes and stepped fringes with complex fringe direction and density variations. In this scenario, the Pivoting median filter, Goldstein filter, Lee filter, and NL-InSAR filter all show a strong dependence on coherence, and the phase error images generated by these four methods clearly show a gradient from black to white. InSAR-BM3D and Φ-Net performed well, but there is still obvious fringe edges information in the phase error images. The proposed method is significantly improved in fringe edges information. 

In Scenario 4 (the seventh and eighth lines in Figure 3), the fringe density is very large and the variation of the fringe direction is complicated, in this scenario, all filtering methods fail to perform well, but relatively speaking, the proposed method has better detail preservation in the low-coherence region and basically recovered the fringe structure correctly. While the filtered image of Φ-Net shows a large number of residues at the top of interferogram, which destroy the whole-period structure of the interference fringes, the performance of Φ-Net is not even as good as InSAR-BM3D. In the phase error images, the proposed method has fewer errors in the fringe structure in the phase error image compared to the other methods.

It can be seen from the above qualitative analysis that the model proposed in this study is optimal. Compared with the existing filtering methods, the proposed method sufficiently suppress noise and preserve detail in areas with low-coherence and dense fringes. The proposed model shows good performance in all four scenarios, which indicate that the proposed model has good generalization ability.

Quantitative evaluation analysis is as follows:

The quantitative evaluation results are presented in Table 1, which shows the average RMSE and SSIM values for the four scenarios, the proposed method achieves the lowest RMSE and the highest SSIM. In terms of efficiency, the advantages of the proposed method are clear, with a prediction time of just 0.14 s per image, which is a hundred to a thousand times more efficient than other methods. The proposed method decreased the huge number of parameters and hardware cost of model training, and improve efficiency in fitting.

In Figure 4, the RMSE and SSIM variation curves are calculated from top to bottom of the interferogram using a sliding window of 32 × 32. The curve trend shows that the performance of all six methods improves as coherence increases. Overall, NL-InSAR, InSAR-BM3D, Φ-Net and the proposed method far outperform the Goldstein, Lee, and pivoting median approaches, as shown by the higher RMSE curves and lower SSIM curves. In the case of the proposed method, the RMSE curve remains largely at the bottom, and the SSIM curve remains largely at the top. As these two curves are less affected by coherence, this indicates that the proposed method’s performance is least dependent on coherence. In line with the qualitative evaluation, the RMSE and SSIM curves illustrate that the proposed method significantly outperforms the other six methods in areas of low coherence.

### 3.3. Experiment Using Real Data

**Figure 5:**
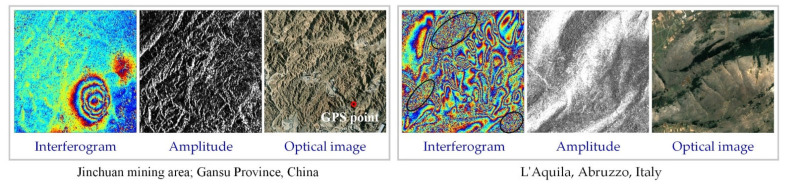
Interferogram, amplitude, and optical images of Jinchuan mining and L’Aquila city. In the Jinchuan mine area, the amplitude image is from the sentinel-1A image on 1 December 2014, the optical image is from google earth’s historical image on 31 December 2014, and the latitude is from 38°27′49″ N to 38°28′58″ N, the longitude is from 102°09′15″ E to 102°10′24″ N. In L’Aquila, the amplitude image is from the COSMO-SkyMed image on 12 April 2009, the optical image is from google earth’s historical image on 8 May 2009, and the latitude is from 42°14′56″ N to 42°15′48″ N, the longitude is from 13°38′30″ N to 13°39′18″ N.

To verify the applicability of the proposed method to real InSAR interferograms, SAR images were selected from different sources (Table 2). The first interferogram was generated from Sentinel-1A data for the Jinchuan Mining Area, Jinchang City, in China’s Gansu Province. The interferogram’s circular interference fringes in pit areas reflect perennial mining operations. The second interferogram was generated from COSMO-SkyMed data for the city of L’Aquila in the western foothills of Italy’s Gran Sasso Mountains. The interferogram’s dense and complex interference fringes are caused by the area’s topographic relief and seismicity, as well as vegetation-covered areas (marked with black ovals in the Figure 5), which are almost completely decoherent and were therefore excluded from the evaluation.

In the real data experiments, noise suppression capability was evaluated by observing the smoothness of the filtered interferogram and the distribution of residues (in non-decoherent regions only). Detail preservation capability can be evaluated by observing the integrity of the interference fringe structure after filtering, as well as resolution preservation, residual structure in noise images.

**Figure 6:**
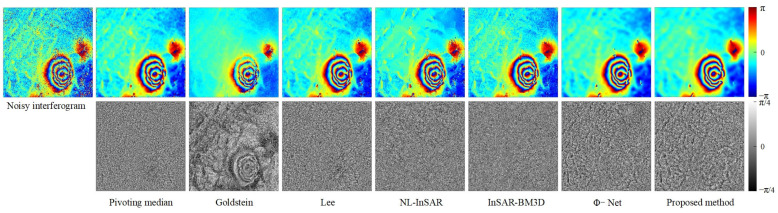
The noisy interferogram filtering results for the Jinchuan mine. The first row shows the filtered interferogram. The second row shows the noise image; the visibility of the phase fringe traces in the noise image indicates the phase filtering method’s ability to preserve detail.

In the case of the Jinchuan mine (Figure 6), the proposed method clearly has the strongest noise suppression capability; the filtered interferogram is smooth, and the fringe is clear and continuous. The Pivoting median filter also performed well in the Jinchuan mine experiments because of the region’s high coherence and stability. However, the Goldstein filter returned severe systematic errors, and the clear fringe traces in the noise image indicate significant loss of useful information. The Lee, NL-InSAR, and InSAR-BM3D filters failed to adequately suppress residues or restore fringe information at the pit. It is also clear that the proposed method left a small number of residual fringe traces in the noise image, indicating over-filtering. The Lee, NL-InSAR, and InSAR-BM3D filters achieved better detail preservation and resolution retention in the region outside the pit, but filtering performance was very limited in the pit region. 

**Figure 7:**
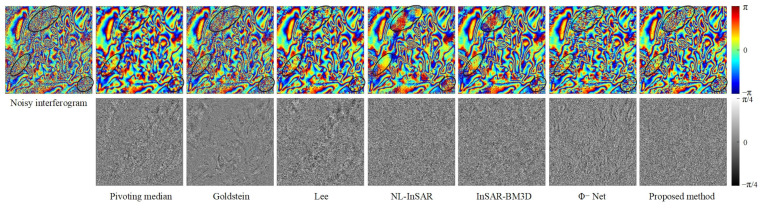
Filtered and unwrapped results for real data from L’Aquila, Italy. The first row shows the filtered interferogram. The second row shows the noise image; the visibility of the phase fringe traces in the noise image indicates the phase filtering method’s ability to preserve detail.

In L’Aquila (Figure 7), the proposed method’s denoising effect was remarkable. In regions free of decorrelation, the interferogram produced by the proposed method exhibits continuous and smooth unwrapping. In contrast to the Jinchuan mine experiments, the proposed method was free of over-filtering in L’Aquila. The corresponding noise images seem closer to Gaussian-distributed white noise, with no obvious residual fringe structure. However, the Pivoting median, Goldstein, and Lee filters clearly exhibit over-filtering.

The proposed method achieved outstanding filtering performance in the real data experiments with different distributions from two different sources. In low-coherence fringe-dense regions, the proposed method efficiently recovered fringe detail information while suppressing noise, demonstrating its generalization capability and application potential.

## 4. Discussion

### 4.1. Performance Robustness of the Model to Coherence in Real Data

**Figure 8:**
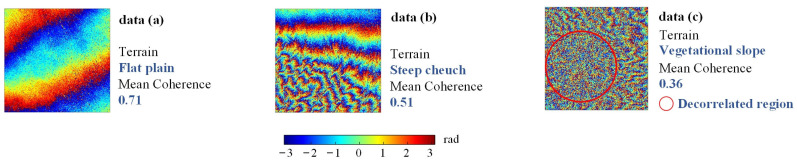
Real interferograms for three different coherence and topographic conditions. (**a**) An interferogram from a flat terrain. (**b**) An interferogram from a steep terrain. (**c**) An interferogram from an area with high vegetation coverage.

In real interferograms, the variation of coherence will not have the regularity of the simulated data in Section 3.2 and will be severely decorrelated due to conditions such as topography and vegetation cover. In this section, we select the real interferograms with different coherence conditions to discuss the adaptability of the methods to the noise intensity and the treatment of decorrelated regions. The Sentinel-1A data of the descending orbital used in this section are from Xinjiang, China, with the master image time of 10 May 2019 and the slave image time of 24 December 2019 After the interference of the two images, the interferogram was randomly cropped, and the interferograms with different coherence conditions were selected as shown in Figure 8.

**Figure 9:**
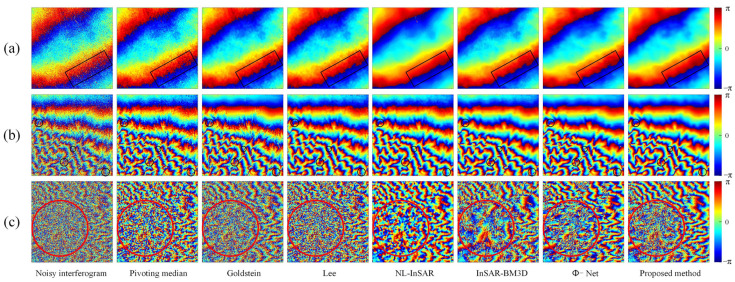
The noisy interferograms and filtered images of real data (**a**–**c**) from Figure 8. (**a**) Flat terrain, (**b**) Steep terrain, (**c**) Areas with high vegetation coverage.

Real data (a) has high coherence and sparse fringes, which is not very challenging for filtering methods. In Figure 9a, all methods yielded complete fringes close to two cycles, however, the Pivoting median filter still did not effectively suppress the residues in data (a), leaving the filtered image still isolated in structure as shown in the rectangular box in Figure 9a. Both the Goldstein and Lee filters showed some degree of resolution degradation, and the edges of the phase were not sharp and not smooth. Compared with the Pivoting median, Lee, and Goldstein filters, the methods of NL-InSAR, InSAR-BM3D, Φ-Net and proposed method perform relatively well in this scenario, with good residues suppression, clear and smooth fringes, the proposed method has a clear advantage in resolution maintenance.

In real data (b), the density and orientation of the interference fringes change rapidly, a condition in which the correct recovery of the fringe structure is crucial. In the black circles in (b), the proposed method tried to recover a complete fringe as much as possible, while Φ-Net shows serious filtering errors in these regions, destroying a whole cycle of the fringe structure, and similarly, NL-InSAR and InSAR-BM3D do not handle these regions as well as the proposed method, even though they outperform Φ-Net.

The real data (c) is a very challenging scenario with an average coherence of only 0.36 and a severe decorrelation inside the red circle. In this scenario, two aspects were compared. Firstly, whether the filtering method can recover complete, clear interference fringes when the fringes are faintly visible outside the red circle. Second, whether the filtering algorithm generates pseudo-fringes under the condition of decorrelation inside the red circle. From the filtered results, it can be found that the integrity and correctness of the restored fringes of the proposed method are better outside the circle, compared to Φ-Net, NL-InSAR and InSAR-BM3D these methods restored the fake fringes in the decorrelated condition inside the red circle, which is obviously unreasonable because it will produce incorrect results in phase unwrapping, which will affect the final InSAR inversion results [34]. Although the Pivoting median, Goldstein, and Lee filters do not generate fake fringes, they do not achieve even the most basic noise suppression in the region outside the red circles, and therefore we believe that these three methods are not sufficiently applicable in this challenging scenario.

In summary, the proposed method is able to cope effectively under the conditions of coherence change, especially under the conditions of extreme low coherence, without either the problem of fake fringes or the problem of insufficient noise suppression.

### 4.2. Evaluation of Filtering Method Performance by Phase Unwrapping

Interferogram filtering directly serves phase unwrapping [35], the continuity or reasonableness of unwrapped images can be used to evaluate the performance of the proposed method. 

**Figure 10:**
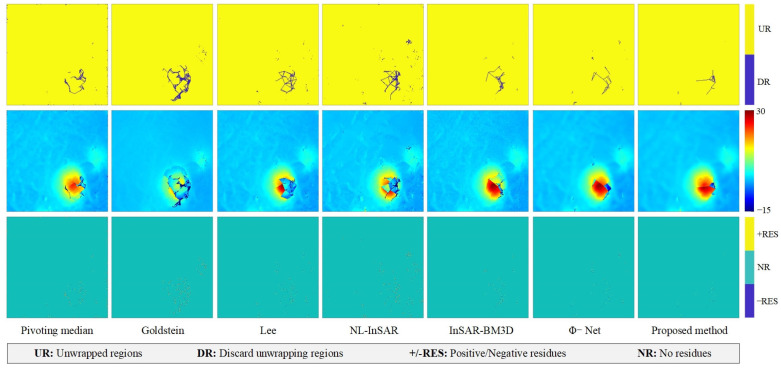
Phase unwrapping results for the case of the Jinchuan mine area in Section 3.3, that the first row is unwrappable area, the second row is unwrapping result. the third row is residues distribution. The unwrapped area image in the first row and the phase unwrapped image in the second row support the indirect evaluation of filtering quality. The residue images in the third row evaluate noise suppression capability.

**Figure 11:**
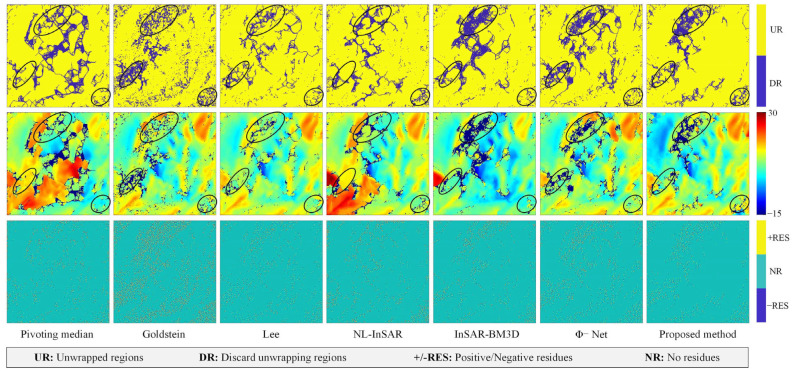
Phase unwrapping results for the case of L’Aquila, that the first row is unwrappable area, the second row is unwrapping result. the third row is residues distribution.

Figure 5 shows the terrain conditions of the real data from the Jinchuan mine area and L’Aquila, Figure 10 and Figure 11 show the unwrapping results of the two terrain conditions. In the case of the Jinchuan mine area, only filtered interferogram from NL-InSAR and the proposed method have a large unwrapped area at the pit. However, in combination with [3,36] and the optical image, the unwrapping result from the proposed method has a more reasonable shape at the pit and is closer to a complete funnel-shaped deformation area. At the same time, in the residues image, the proposed method captured the smallest number of residues, indicating that the proposed method has the best detail preservation in this case.

The example of L’Aquila is very challenging for phase unwrapping because there are large decorrelation regions in the image, and in reality, it is difficult to monitor these regions using the InSAR technique; therefore, in the experiments of this paper, we believe that the unwrapping results in this region are not realistic (inside the black ovals in Figure 11), and therefore do not expect these regions to be unwrapped. In the regions outside the oval markers, we expect continuous, smooth unwrapped results by phase unwrapping due to the absence of complete decorrelation [34]. After comparison, the unwrapped results of the proposed method have better continuity, while the other methods show a large number of discontinuities in the regions outside the oval markers, which is obviously unreasonable based on the actual terrain conditions in the Figure 5. Moreover, the Pivoting median, Goldstein, Lee, and NL-InSAR filters all perform the unwrapping within the black ovals, which is not consistent with this actual condition, indicating that these filtering methods have pseudo-information problem in the decorrelated regions.

### 4.3. Evaluation of Filtering Methods Using TS-InSAR

**Figure 12:**
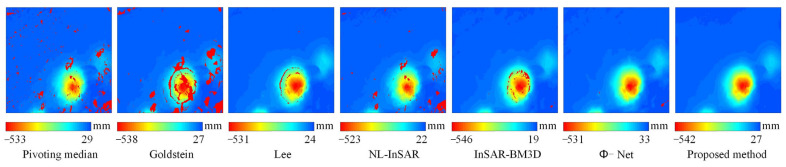
The cumulative settlement from SBAS-InSAR.

Phase filtering has a key influence on the measurement results of TS-InSAR, in this section, we selected 16 scenes of sentinel-1A images as shown in Table 3, the other image parameters are the same as in Section 3.3, and the cumulative settlement of the Jinchuan mine area from 14 October 2014 to 7 January 2017 were obtained using small baseline subset (SBAS)-InSAR technique as shown in Figure 12 [37,38]. In multiple experiments, the filtering methods were used separately as mentioned in the paper, and other parameters were kept consistent.

**Figure 13:**
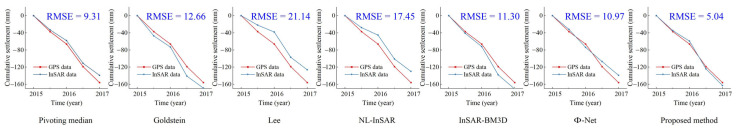
Comparison of InSAR results and GPS data for validation.

In the Figure 12, combining the experimental results in the literature [3,39] and the real terrain conditions in the Figure 5, the proposed method obtains more complete and reasonable deformation results, while the deformation results obtained by Pivoting median, Goldstein and NL-InSAR filters show large null areas (the white area in the Figure 12), and the deformation results obtained by Lee, InSAR-BM3D and Φ-Net have small null areas. To further evaluate the effect of different filtering methods on the time-series deformation, Global Positioning System (GPS) data (the location of the GPS point is marked in the optical image in Figure 5) are used to verify the advantages of the proposed method in terms of deformation accuracy [39]. From the Figure 13, it can be found that the proposed method obtains the closest results to the GPS data, the RMSE of proposed method is only 5.04, indicating that the proposed method has obvious application advantages.

### 4.4. Existing Problems and Future Research

As CNN-based interferogram filters are highly data-driven, training sample production is crucial. Generative adversarial networks (GAN) can generate high-fidelity simulated images (unsupervised or conditionally) to improve the model’s performance and generalization [40], and we will use GAN in future studies to generate more diverse and realistic terrain data for synthesizing interferogram samples.

In addition, the strategy of using multi-level convolutional neural networks certainly provides significant improvement in detail preservation and noise suppression, however, multi-level feature fusion also has the possibility of feature redundancy. Therefore, the embedding of an attention mechanism in the model is also an effective way to improve the performance and efficiency of the model, which we will consider to in subsequent research.

## 5. Conclusions

In this study, the proposed CNN-based interferogram filtering method achieved better filtering performance and higher computational efficiency than current phase methods. The study used mathematical fractal methods and real DEM to obtain simulated datasets. On the input and output sides of the model, real phases were converted to complex model phases to avoid the problem of noise discrimination at phase edges. A convolutional neural network model with multi-scale features was then constructed using a multi-path multi-level feature fusion strategy. This approach enabled the network to take account of multi-scale semantics, enhancing noise suppression and detail preservation for noiseless interferogram prediction. Finally, the proposed method’s performance was evaluated subjectively and objectively.

In the experiments based on simulated data, the proposed method’s superior performance persisted as coherence changed. Filtered images were self-adaptive in regions of differing coherence, with no over- or under-filtering. Filtering time was very efficient at 0.14 s, and RMSE and SSIM values reached 0.74 and 0.96, respectively. The proposed method’s applicability was confirmed in the experiments based on real data, matching performance in the simulated data experiments in terms of efficient noise suppression and detail preservation under different conditions. The unwrapped images of real data show that the proposed method captured the most reasonable unwrapped shape; in regions free of decoherence, the method captured almost the maximum unwrapped area. In the TS-InSAR experiments, the proposed method obtains the most complete time-series deformation results and is closest to the GPS data. The results indicate that the proposed method is applicable to InSAR interferogram filtering and is superior to commonly used interferogram filtering methods in terms of performance and efficiency.

## Figures and Tables

**Table 1:** Quantitative evaluation for simulated interferogram filtering.

Method	RMSE	SSIM	T(s)
Pivoting median	1.38	0.83	3.5
Goldstein	1.73	0.76	10.4
Lee	1.35	0.85	11.6
NL-InSAR	0.95	0.91	168.1
InSAR-BM3D	0.88	0.93	22.1
Φ-Net	0.84	0.93	0.21
Proposed Method	0.74	0.96	0.14

**Table 2:** Parameters of the real data.

Case	Platform	Time of Master Image	Time of Master Image	Polarization	Orbital Direction	Band
Jinchuan mine area	Sentinel-1A	1 December 2014	18 May 2015	VV	Ascending	C
L’Aquila	COSMO-SkyMed	12 April 2009	14 May 2009	HH	Ascending	X

**Table 3:** SBAS-InSAR data selection.

Sequence	Time	Sequence	Time
1	14 October 2014	9	20 December 2015
2	1 December 2014	10	6 February 2016
3	11 February 2015	11	25 March 2016
4	31 March 2015	12	5 June 2016
5	11 June 2015	13	16 August 2016
6	29 July 2015	14	3 October 2016
7	9 October 2015	15	20 November 2016
8	26 November 2015	16	7 January 2017

## Data Availability

The data are not publicly available as they involve the subsequent application of other studies.
